# The CodY regulator is essential for virulence in *Streptococcus suis* serotype 2

**DOI:** 10.1038/srep21241

**Published:** 2016-02-17

**Authors:** Liping Feng, Jiawen Zhu, Haitao Chang, Xiaoping Gao, Cheng Gao, Xiaofeng Wei, Fangyan Yuan, Weicheng Bei

**Affiliations:** 1 State Key Laboratory of Agricultural Microbiology, College of Veterinary Medicine, Huazhong Agricultural University, Wuhan 430070, China; 2 Shanghai Laboratory Animal Research Center, Shanghai 201203, China; 3 Hubei key laboratory of Animal Embryo and Molecular Breeding, Institute of Animal Husbandry and Veterinary Sciences, Hubei Academy of Agricultural Sciences, Wuhan 430070, China; 4 Cooperative Innovation Center of Sustainable Pig Production, Wuhan 430070, China

## Abstract

The main role of CodY, a global regulatory protein in most low G + C gram-positive bacteria, is in transcriptional repression. To study the functions of CodY in *Streptococcus suis* serotype 2 (*S. suis* 2), a mutant *codY* clone named *∆codY* was constructed to explore the phenotypic variation between *∆codY* and the wild-type strain. The result showed that the *codY* mutation significantly inhibited cell growth, adherence and invasion ability of *S. suis* 2 to HEp-2 cells. The *codY* mutation led to decreased binding of the pathogen to the host cells, easier clearance by RAW264.7 macrophages and decreased growth ability in fresh blood of *Cavia porcellus*. The *codY* mutation also attenuated the virulence of *S. suis* 2 in BALB/c mice. Morphological analysis revealed that the *codY* mutation decreased the thickness of the capsule of *S. suis* 2 and changed the surface structures analylized by SDS-PAGE. Finally, the *codY* mutation altered the expressions of many virulence related genes, including sialic acid synthesis genes, leading to a decreased sialic acid content in capsule. Overall, mutation of *codY* modulated bacterial virulence by affecting the growth and colonization of *S. suis* 2, and at least via regulating sialic acid synthesis and capsule thickness.

*Streptococcus suis* (*S. suis*) is a prevalent pathogen and an important zoonotic agent in swine. *S. suis* causes great economic losses to the swine industry worldwide each year and is responsible for a variety of diseases, including meningitis, septicemia, arthritis, pneumonia, and even death^1^. Based on the capsular polysaccharides, 33 serotypes (types 1 to 31, 33, and 1/2) have been identified^2^. Among these serotypes, *S. suis* 2 is thought to be the most virulent and is the most frequently isolated in association with diseases in most countries^2^. *S. suis*, especially serotype 2, also causes serious infections in humans who are in contact with infected swine or pork-derived products^3^,^4^ and can induce meningitis, septic shock and permanent hearing loss^4^,^5^. Hitherto, there are many cases of human *S. suis* infection have been reported in China, United States, Canada, New Zealand, Australia Korea and Greece^6^,^7^,^8^,.

Although many studies of *S. suis* 2 have been published in recent years, our understanding of the pathogenesis and virulence factors remains very limited^9^. The virulence of *S. suis* is associated with the polysaccharide capsule, which is rich in sialic acid and confers antiphagocytic properties on *S. suis*. In murine and porcine infection models, the *S. suis* capsule is a virulence factor because capsular mutants are nonpathogenic and are more rapidly cleared from the bloodstream than the wild-type strain^10^. The virulence of *S. suis* is also associated with the pathogen-host interaction. Muramidase-released protein (MRP) is as an important virulence marker of *S. suis* 2. MRP can bind human fibrinogen (hFg) and the MRP-hFg interaction increases the permeability of the blood-brain barrier (BBB) through which regulating the development of *S. suis* meningitis^11^. Rgg as a global transcriptional regulator, promotes S. suis 2 bacterial survival during pathogen-host interaction^12^. In the infected host, *S. suis* uses the two-component system (TCS) as the common regulatory mechanism to response to environmental signals. The TCS includes at least 15 components that have been predicted through bioinformatics analysis^13^. Besides TCSs, *S. suis* also uses other regulators, such as CcpA^14^ and Zur^15^, to respond to changing environments.

CodY is a global transcriptional regulator of gene expression in low G + C gram-positive bacteria^16^. In *Bacillus subtilis*, CodY regulates metabolic pathways and cellular processes, such as peptide and sugar uptake, branched-chain amino acid (BCAA) biosynthesis, the development of genetic competence and the regulation of cellular motility^17^. CodY also regulates primary metabolism like carbon metabolism, iron uptake and biosynthesis of branched-chain amino acids in other bacterias including *Streptococcus pneumonia*^18^*, Streptococcus mutans*^19^, and *Streptococcus thermophiles*^20^. In addition, CodY sensed the oligopeptide permease Ami to repress competence for genetic transformation in *Streptococcus thermophilus*,^21^. Recently, in *Streptococcus pyogenes*, CodY is reported to alter the abundance of a select group of exoproteins, including DNases, a protease, and hylauronidase, which may alleviate starvation by promoting dissemination of the pathogen to nutrient rich environments and by hydrolysis of host macromolecules^22^.

Besides regulating genes involved in primary metabolism, CodY also regulates the virulence mechanism in various pathogenic gram-positive bacteria^23^. CodY represses the synthesis of many virulence factors of *Staphylococcus aureus*. The introduction of a *codY*-null mutation into two *S. aureus* strains resulted in the overexpression of several virulence genes. The *codY*-null strains had higher hemolytic activities, produced more polysaccharide intercellular adhesin (PIA), and formed more robust biofilms than did their isogenic parent strains. And *codY* mutation in *S. aureus* induced hyper-production of secreted proteases, leukocidins and hemolysins^24^. CodY also positively regulates bacteria virulence. It activates toxin gene expression by regulating the global regulator AtxA in *B. anthracis*. The disruption of CodY did not affect either *ex vivo* or *in vivo* capsulation, but led to attenuated virulence of a wild-type *B. anthracis* strain in a mouse model of infection^25^. In *Streptococcus pyogenes*, CodY and CovRS act in opposite directions, with CodY stimulating and CovRS repressing a substantial fraction of the core genome, including many virulence factors^26^. CodY also regulates pel/sagA and mga, loci that themselves positively affect the expression of numerous virulence factors in growth phase-dependent way in *Streptococcus pyogenes*^27^. Malke *et al*. found that CodY could indirectly affect the transcription of the majority of virulence genes^28^. Thus, in terms of virulence, CodY has a variety of roles that might be species-dependent.

Pathogenic bacteria possess a large arsenal of surface organelles and secreted toxins that allow them to occupy many different niches throughout the course of infection. Successful infection by bacterial pathogens requires adhesion to host cells, colonization of tissues, and in certain cases, cellular invasion – followed by intracellular multiplication, dissemination to other tissues, or persistence^29^. In mouse models, *codY* is found to be transcribed in the murine nasopharynx and lungs and is specifically required for colonization. The finding was underscored by the diminished ability of the *codY* mutant to adhere to nasopharyngeal cells *in vitro*^18^. Further study showed that CodY activates pcpA, regulating the adherence to nasopharyngeal cells, suggesting a direct link between nutritional regulation and adherence *in Streptococcus pneumoniae*^18^.

In this study, we used an *in vitro* model as reported^1^,^30^ to compare the adhesion ability of Δ*codY* strain to mammalian cells with that of the wild-type. We further evaluated other phenotypic changes induced by mutation of *codY* in *S. suis* 2. Mutation of *codY* significantly decreased the growth rate, adherence and invasion ability *in vitro*, and capsule thickness of *S. suis* 2. The *codY* mutation also reduced the virulence of *S. suis* 2 in BALB/c mice and enhanced phagocytosis of *S. suis* 2 mediated by macrophages. Using a microarray assay and the real time PCR, we further demonstrated that *codY* mutation inhibited the expression of sialic acid synthesis gene, which correlated with the observation that *codY* mutation decreased the cellular sialic acid content. And the analysis through SDS-PAGE revealed that *codY* mutation changed the surface structures of *S. suis* 2. In general, this study would enhance our understanding of virulence regulation mediated by CodY in *S. suis 2*.

## Results

### The *codY* mutation inhibited cell growth of *S. suis* 2

**Figure 1:**
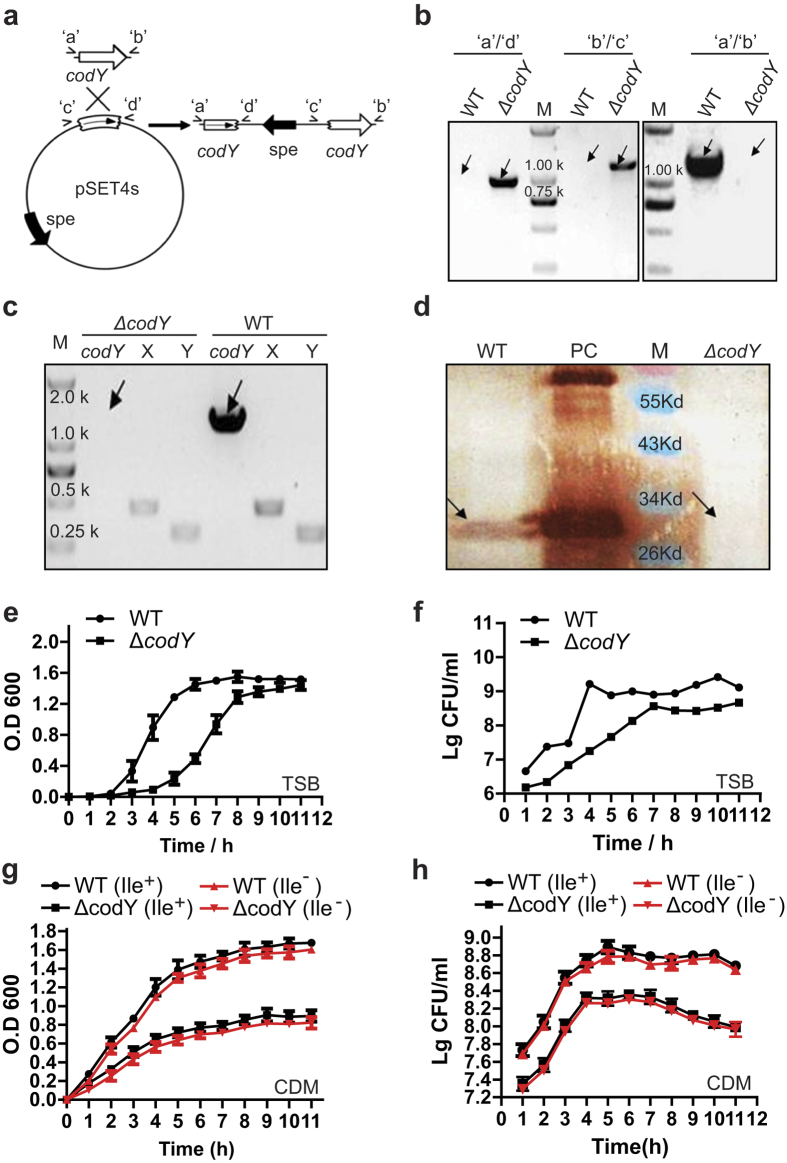
Construction of the *codY* mutant strains and growth curves of the wild-type (WT) and ∆*codY* strains. (**a**) Strategy for the mutation of *codY* in *S. suis* SC19 by homologous recombination. PCR was used to amplify the *codY* DNA fragment, which was cloned into the pSET4s vector to produce pSET4s-*codY’* which was used for the *codY* gene insertion mutation. ‘**c**’ and ‘**d**’ indicate the left and right homologous arms of *codY*. (**b**) PCR confirmation of the mutant strains. The primer pairs used in the PCR analysis are indicated above the lanes. Genomic DNAs from the wild type (WT) and *codY* mutation (∆*codY*) strains were used as templates. (**c**) RT-PCR confirmed that the only WT expressed the *codY* mRNA. (**d**) Western blotting was used to confirm that the expression of CodY (predict size of 29.3 kDa) was disrupted in the ∆*codY* strain. M, the Pre-stained protein marker with the indicated sizes; an arrow shows the predicted CodY band position. PC, Positive Control, purified CodY protein expressed by *E. coli* BL21(DE) from vector pET-28a-*codY*-His. (**e–h**) Effect of the *codY* mutation on the growth of *S. suis* 2, which was evaluated by OD 600 and CFU counting at indicated time points in TSB medium with isoleucine (Ile^+^) (**e,f**) or in the chemically defined medium (CDM) with (Ile^+^) or without isoleucine (Ile^−^) (**g,h**), respectively. WT (Ile^+^) and WT (Ile^−^) , wild type strain cultured in CDM with or without isoleucine; ∆*codY* (Ile^+^) and ∆*codY* (Ile^−^)*, codY* mutation strain cultured in CDM with or without isoleucine. Each curve shown is representative of a typical experiment that was performed three times. Each curve shown isrepresentative of a typical experiment that was performed three times.

To investigate virulence regulation by *codY* in *S. suis* 2, we constructed a mutant strain for *codY*, termed ∆*codY*, in the SC19 wild-type strain obtained from Sichuan Province by inserting a long sequence into the *codY* gene (Fig. 1a). Using the genome of SC19 as the template, the DNA fragment *codY’* was amplified by PCR before being cloned into the pSET4s vector to produce pSET4s-*codY’*. Using homologous recombination, pSET4s-*codY’* was inserted into the endogenous *codY* gene to disrupt the functional expression of CodY (Fig. 1a). The ∆*codY* strain was verified by PCR analysis (Fig. 1b) combined with DNA sequencing and reverse transcription PCR (RT-PCR) analysis (Fig. 1c). Western blotting confirmed that the expression of CodY was lost in the ∆*codY* strain compared to that of parental strain SC19 (Fig. 1d).

We then investigated the effect of the *codY* mutation on the basic biological properties of *S. suis* 2. In solid medium, ∆*codY* strains formed smaller colonies compared with the wild-type (WT) strain. And in further growth curve analysis of *S. suis* 2, mutation of *codY* caused a growth flaw either in Tryptic Soy Broth (TSB) medium containing isoleucine (Fig. 1e,f), in chemically defined medium^31^ with isoleucine or without isoleucine (Fig. 1g,h).

### The *codY* mutation inhibited the adherence and invasion ability of *S. suis* 2

**Figure 2:**
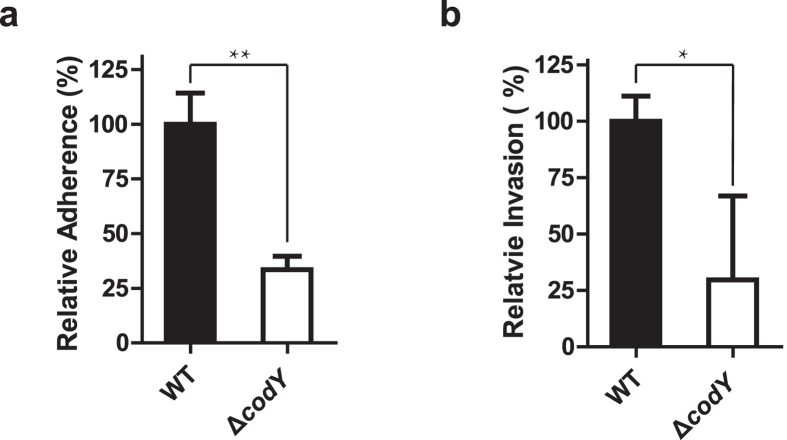
Effect of the *codY* mutation on the adherence and invasion ability of *Streptococcus suis* serotype 2. (**a,b**) Evaluating the effect of *codY* mutation on adherence and invasion ability of *S. suis* 2. The 10^5^ HEp-2 cells maintained in 24-well plate were infected with *S. suis* 2 (10^6^ CFU) at a MOI (multiplicity of infection) of 10:1 and incubated at 37 °C for 2 h. The infected cells were washed 3 times with PBS and lysised by sterile distilled water followed by plating the lysis on TSA for cultivation in the 37 °C incubators for 24 hours, determining the CFU numbers of bacteria adhered on HEp-2 cells in indicated groups. The adherence experiments were repeated at least three times (**a**). The *S. suis 2* (10^6^ CFU) at a MOI of 10:1 was incubated with 10^5^ HEp-2 cells at 37 °C for 2 h. The infected cells were washed 3 times with PBS before being kept in DMEM culture medium containing gentamicin (100 μg/ml) and penicillin (5 μg/ml) for 2 h to kill the bacteria outside the HEp-2 cells. Then the HEp-2 cells were washed thrice by PBS followed by calculating the CFU by plating the lysis of the cells for thrice in indicated groups. The percentage of the CFU was normalized to WT group designed as 100%. The data was showed as Means ± S.E.M; The statistic was conducted using Student’s *t* test (unpaired *t* test, two tailed); **P < 0.01; *P < 0.05 (**b**).

Successful establishment of infection by pathogens requires adhesion to host cells, colonization of tissues, and in certain cases, cellular invasion followed by intracellular multiplication, dissemination to other tissues, or persistence^29^. Thus, we used an *in vitro* model using HEp-2 cells to evaluate the effect of *codY* mutation on the adhesion ability of *S. suis* 2 to mammalian cells^1^. Firstly, we compared the adhesive ability of ∆*codY* strain to mammalian cells with that of the WT. The result revealed that the *codY* mutation caused a significant reduction (n = 3, *P* < 0.01) in adherence to human laryngeal epidermoid carcinoma HEp-2 cells compared to the WT (Fig. 2a). The effect of the *codY* mutation on the invasion into mammalian cells was also evaluated. The result indicated that the *codY* mutation induced a significant decrease (n = 3, *P* < 0.05) in cell invasion into HEp-2 cells compared with WT (Fig. 2b). These *in vitro* results revealed that the *codY* mutation could decrease the virulence of *S. suis* 2 through attenuating the successful establishment of infection on host mammalian cells.

### The effect of the *codY* mutation on anti-phagocytosis and anti-killing effect against macrophages, and its growth ability in mammalian blood

**Figure 3:**
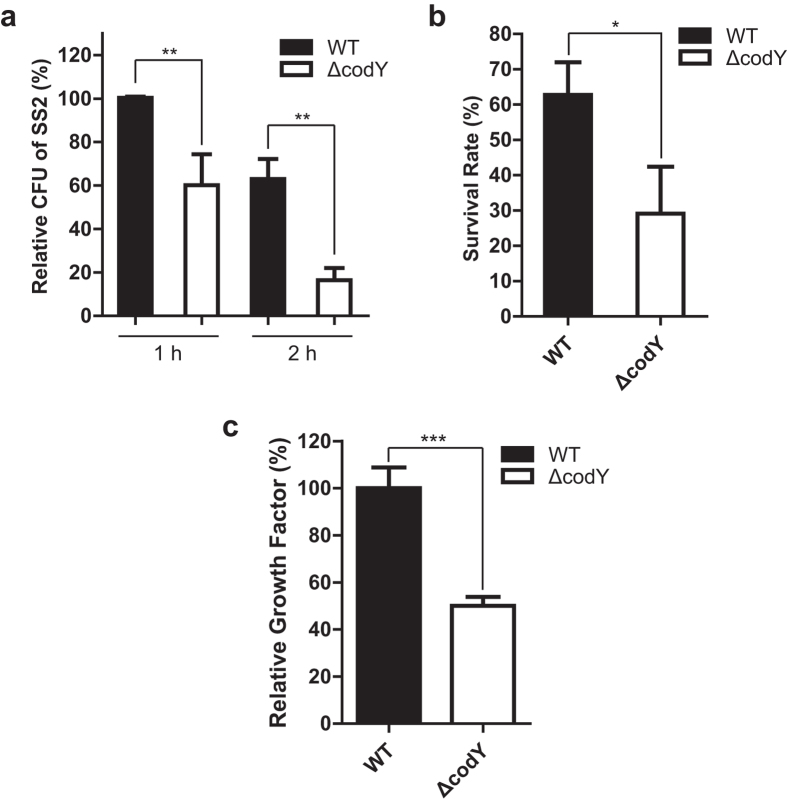
Effect of *codY* mutation on phagocytosis and killing of pathogens by macrophages and the whole blood. (**a,b**) Attenuated phagocytosis but enhanced killing of the Δ*codY* strain by RAW264.7 macrophages. The wild type (WT) and *codY* mutated (Δ*codY*) strain were incubated with RAW264.7 macrophages (MOI = 100:1) for 1 h followed by washing with PBS and then incubated in DMEM media containing ampicillin (100 μg/ml), when the time point is 0 h, to kill the bacteria cells outside of macrophages. After 1 or 2 hour of incubation, when the time point is 1 h or 2 h, the macrophages were washed, lysed with water followed by plating the lysate dilutions on TSB agar and determining the formed colonies associated to 1 h and 2 h time point respectively. The percentage of the CFU was normalized to WT group at 1 h designed as 100%. The data was showed as Means ± S.E.M; The statistic was conducted using Student’s *t* test (unpaired *t* test, two tailed); **P < 0.01 (**a**); The rates of the survival for WT strains and Δ*codY* ones were compared. The survival rate was calculated as CFU at 2 h/ CFU at 1 h × 100%. Each assay was performed in triplicate (**b,c**) Growth factors of the WT and Δ*codY* strains in pig blood. Approximate 10^6^ CFU of the WT and Δ*codY* strains were incubated in heparinized pig blood and incubated for 2 h at 37 °C with end-to-end rotation. Growth factor was defined as the ratio of CFU in each sample after 2 h incubation over the CFU in the corresponding inoculum. Each assay was performed in triplicate. The data are expressed as the means ± *S.D*. of three independent experiment; ***P < 0.001; **P < 0.01; *P < 0.05.

We further used the RAW264.7 macrophages to evaluate the effect of the *codY* mutation on pathogen virulence through comparing the macrophage mediated phagocytosis and killing of WT and *codY* mutation strains. The result showed that the RAW264.7 cells phagocytosed significantly more WT strains than ∆*codY* ones in 1 h (n = 3, *P* < 0.01) and 2 h (n = 3, *P* < 0.01) (Fig. 3a), respectively. This decreased phagocytosis of ∆*codY* strain could be caused by the attenuated adherence induced by *codY* mutation. Though both the CFU of WT and ∆*codY* strains decreased which caused by the clearance effect inside the RAW264.7 cells with the time went on (Fig. 3a), the rate of the survival for WT strains is significantly (n = 3, *P* < 0.05) higher than that of the ∆*codY* ones (Fig. 3b). This result indicated that the *codY* mutation decreased the virulence and led to the cells easier being cleared by RAW264.7 macrophages. In subsequent bactericidal assays, the growth ability of ∆*codY* strain was also significantly lower in fresh blood of *Cavia porcellus* than that of WT strain in 2 h (Fig. 3c).

Taken together, the *in vitro* results revealed that the *codY* mutation led to the attenuated binding of the pathogen to the host cells, easier clearance by RAW264.7 macrophages and decreased growth ability in fresh blood of *Cavia porcellus*.

### The effect of *codY* mutation on the pathogenicity of *S. suis* 2 in mice

To investigate the pathogenicity of *S. suis* 2 *in vivo*, we assessed the role of *S. suis* 2 CodY protein in the virulence and pathogen growth capacity in tissues by infection of BALB/c mice. Firstly, we established that the maximum non-lethal dose (Dn) for the WT and ∆*codY S. suis* 2 were 1.00 × 10^9^ CFU and 4.56 × 10^9^ CFU, respectively. We then identified the lowest lethal dosage for WT and ∆*codY* strain as 3.91 × 10^6^ CFU and 2.85 × 10^8^ CFU, respectively. Using method of logarithmic spacing, the LD_50_ of the WT and ∆*codY* strain for mice were calculated as 2.57 × 10^8^ CFU and 1.46 × 10^9^ CFU, respectively. Thus, according to the LD_50_ value, the virulence of the *∆codY* strain was decreased when compared with that of the WT.

**Figure 4:**
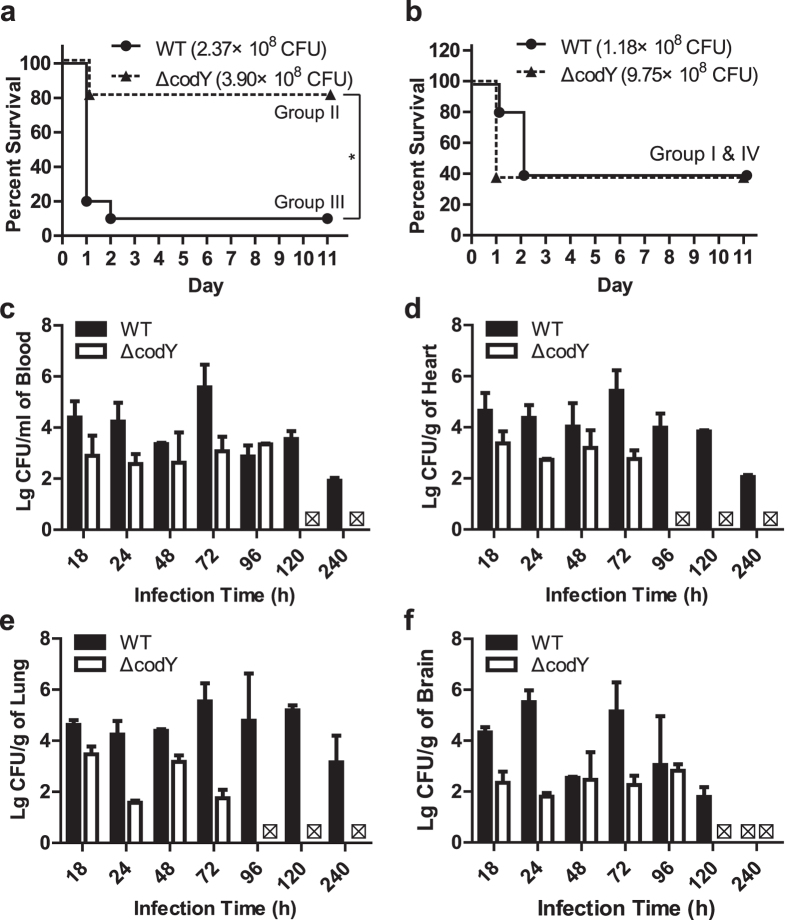
Effect of *codY* mutation on the pathogenicity of *Streptococcus suis* serotype 2 in mice. (**a,b**) Survival curves of mice infected with *S. suis* strains investigated in group I, II, III and IV (see the ‘Experimental procedures’). Groups of ten female BALB/c mice were inoculated with 2.37 × 10^8^ CFU/mouse for the wild-type (WT, ●, group III) or 3.90 × 10^8^ CFU/mouse for the *codY* mutated strain (Δ*codY*, ▲, group II) (**a**), and with 1.18 × 10^8^ CFU/mouse (WT, ●, group I) or 9.75 × 10^8^ CFU/mouse (Δ*codY*, ▲, group IV) (**b**), respectively. Survival data were analyzed with the log-rank (Mantel-Cox) test. Survival was monitored over an 11-day period. (**c–f**) The clearance of pathogen in mouse tissues in infection of BALB/c. The WT and mutated *codY* strains were separately inoculated into 30 mice at 5 × 10^7^ CFU/mouse. Then 3 mice were sacrificed followed by analyzing the amount of bacteria in blood (**c**), heart (**d**), lung (**e**) and brain (**f**) at each indicated time points. The amounts of bacteria were expressed as ‘lg (CFU/g)’ or ‘lg(CFU/ml)’ in each tissue. 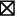, representing the CFU is 0 (no logarithm).

We then investigated the role of CodY in the virulence of *S. suis* 2 using a murine infection model. In the similar inoculum dose groups (Group III and Group II, see ‘Methods’ section), mice infected with the WT developed typical clinical symptoms of *S. suis* 2 infection, including rough coat hair, limping, lethargy and swollen eyes. And for the WT strain, the dose of 2.37 × 10^8^ CFU/mouse (Group III) induced 90% of the mice dead in 48 h (Fig. 4a). In contrast for *∆codY* strain, the dose of 3.90 × 10^8^ CFU/mouse (Group II) only caused 20% mice dead (Fig. 4a). The survival rates were significantly lower (*P* = 0.0247) in mice infected with the WT strain than in that infected with the *∆codY* strain (Fig. 4a). Further, we found that the much higher dose of ∆*codY* strain at 9.75 × 10^8^ CFU/mouse (Group IV) induced similar death rate with that of WT strain at dose of 1.18 × 10^8^ CFU/mouse (Group I) (Fig. 4b). All mice which were inoculated with PBS remained healthy. The results in Fig. 4a,b revealed that the *codY* mutation greatly decreased the virulence of *S. suis* 2 according to the decreased lethal ability in mice.

In addition, we evaluated the pathogen growth capacity in tissues of infected BALB/c mice. The WT and mutated strains were separately inoculated into 30 mice at 5 × 10^7^ CFU followed by analyzing the amount of viable bacteria in blood, heart, lung and brain at time points of 18, 24, 48, 72, 96, 120 and 240 h respectively (Fig. 4c–f). The results showed that the *∆codY* strain started to be eliminated within 24 h. Notably, the amounts of viable *∆codY* strain in the analyzed tissues were 1–4 logarithmic spaces lower than that of the WT at 24 h (Fig. 4c–f), which indicated that *codY* mutation promoted the clearance in blood or tissues of the infected mice. At 96 h or 120 h, the *∆codY* strain was completely eliminated in the different tissues, while the WT remained viable in the different tissues at the corresponding time points (Fig. 4c–f).

Taking together, the survival rates experiment *in vivo* and the assay of growth capacity in the tissues of the infected mice indicated that *codY* mutation could decrease the virulence of the bacteria.

**The effect of** ***codY*** **mutation on the hemolytic activity, capsule thickness and surface structures of** ***S. suis*****2**.

To investigate how the *codY* mutation attenuated the adherence, invasion and virulence of *S. suis* 2 *in vivo*, we further analyzed the effect of *codY* mutation on the hemolytic activity and capsule thickness of *S. suis* 2. Hemolysin is considered as an important virulence factor in *S. suis* 2, because it allows *S. suis* 2 to intrude into deeper tissues and subsequently contributes to the multiplication of *S. suis* 2 in those tissues^32^. Thus, we compared the hemolytic activity of the ∆*codY* strain with that of the WT. Firstly, the WT and ∆*codY* strains were inoculated on Tryptic Soy Agar (TSA) agar containing 5% fresh sheep blood and incubated for 24 hours at 37 °C. The result showed that ∆*codY* strain had the similar hemolytic phenotype as the WT strain. The hemolytic activity is regulated by genes including suilysin (sly), hemolysin III homolog, putative hemolysin and pneumolysin (ply)^13^. Though suilysin expression is decreased in microarray data, we also noticed that the putative hemolysin-III-related protein (SSU05_0925), which is a novel exogenous hemolysis-related gene in *S. suis* 2 strains, is increased in the ∆*codY* strain in the microarray assay (Table 1S). This might be the reason why we did not find the hemolytic activity is changed by *codY* mutation.

**Figure 5:**
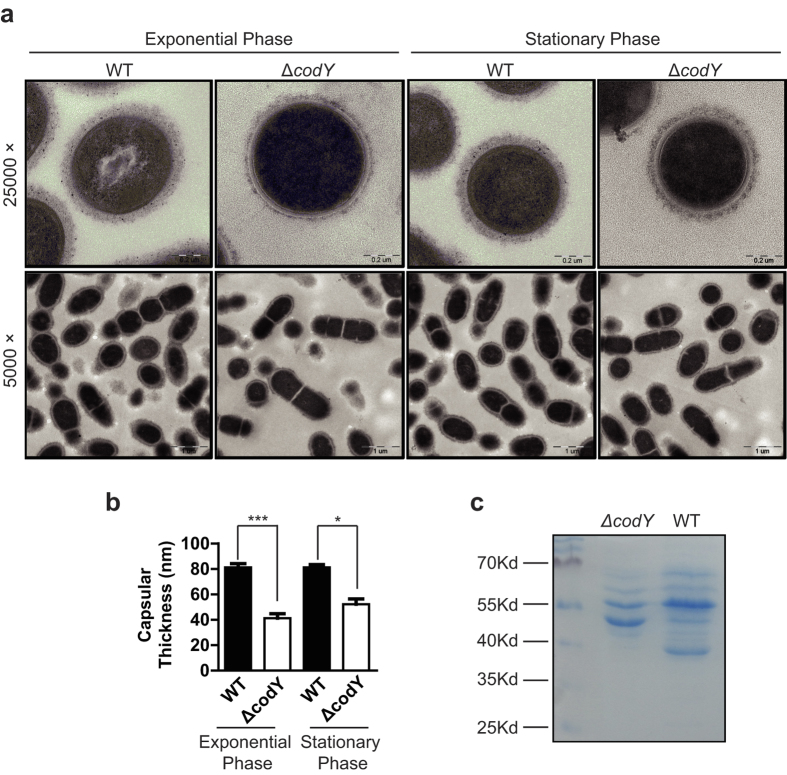
Analyzing cell morphology by transmission electron microscopy and bacterial surface structures by SDS-PAGE. (**a,b**) Transmission electron micrographs of *S. suis* strains. Bars in upper (magnification is 25000) and lower panels (magnification is 5000) were200 and 1000 nm respectively. Bacteria were cultured in TSB containing 10% fetal bovine serum (**a**). Measurement of capsule thickness as described in ‘Method’ during exponential phase (O.D_600_ = 0.7 ~ 1.0) revealed that the thickness of capsules for wild type (WT) and *codY* mutated (Δ*codY*) strains were 81.23 ± 2.91 nm and 41.27 ± 3.41 nm respectively. The capsule thickness during stationary phase (O.D_600_ ≥ 1.2) showed that the thickness of capsules for wild type (WT) and *codY* mutated (Δ*codY*) strains were 81.16 ± 2.22 nm and 52.28 ± 4.06 nm respectively. (**b**). The data are expressed as means ± *S.D*. The means of two groups were compared using Student’s *t* test (unpaired, 2-tailed). ***P < 0.001; *P < 0.05 (**c**) SDS-PAGE analysis of the bacterial surface-associated proteins. The bacteria grown were re-suspended in 1 ml of 10 mM sodium phosphate (pH 5.5), pelleted by centrifugation at 13,000 × g for partially remove proteins not associated with cell surface structures, and then resuspended in 0.2 ml of 10 mM sodium phosphate (pH 5.5). The bacterial suspension was pushed through a 25 G needle four to five times to shear the surface structures and proteins from the bacteria, and then centrifuged at 13,000 × g. 20 μl of the supernatant was used for SDS-PAGE analysis.

One possible explanation for the differences in virulence was *via* an effect on the capsule, whose synthesis could be differentially regulated in the WT and ∆*codY* strain. Using the electron microscopy, we observed that the capsules of the ∆*codY* strain were thinner than those of WT strain during exponential phase and stationary phase (Fig. 5a). During exponential phase (O.D_600_ = 0.7 ~ 1.0), the capsule thickness for wild type (WT) and *codY* mutated (Δ*codY*) strains were 81.23 ± 2.91 nm and 41.27 ± 3.41 nm respectively. And during stationary phase (O.D_600_ ≥ 1.2), the capsule thickness for wild type (WT) and *codY* mutated (Δ*codY*) strains were 81.16 ± 2.22 nm and 52.28 ± 4.06 nm respectively (Fig. 5b). The statistical result showed that the average capsular thickness of the ∆*codY* strain was significantly decreased compared with that of the WT in both exponential phase and stationary phase (Fig. 5b).

Further using SDS-PAGE, we compared the surface structures of *S. suis* 2. The result showed that compared with the WT, the *codY* mutation resulted in decreases in the abundances of proteins of approximately 67 and 55 kDa, an increase in the presence of a protein at 47 kDa and the disappearance of a protein at approximately 40 kDa (Fig. 5c) on the surface structures of *S. suis* 2.

According to these data, the *codY* mutation could attenuate the adherence, invasion ability and virulence of *S. suis* 2 *in vivo* by, at least in part, altering the capsular thickness and surface structures of *S. suis* 2.

### Identification of CodY-regulated genes *via* Microarray and qRT-PCR

**Figure 6:**
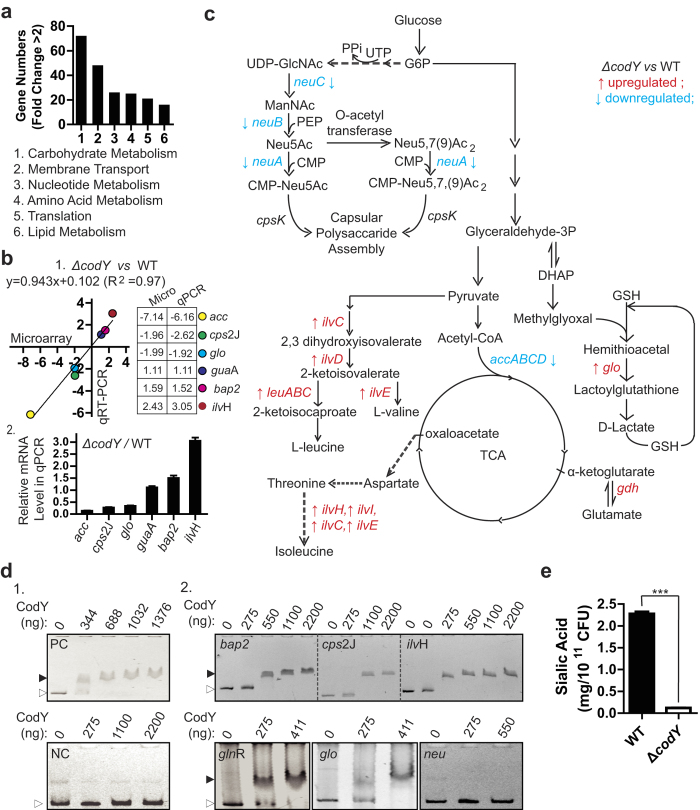
Investigation of *codY*-regulated genes in *S. suis* strain. (**a**) The main signal pathways affected in the *codY* mutation strain. Numbers of genes in the indicated signal pathways whose expression changed by more than 2-fold in response to the *codY* mutation in the microarray assay. (**b**) Six representative gene expressions from microarray (x-axis) and qRT-PCR (y-axis) in wild type (WT) and *codY* mutated (Δ*codY)* strains. 1, The fold changes (Δ*codY VS* WT) of 6 indicated genes from microarray and qRT-PCR showed strong correlations. A linear equation between fold changes from microarray and that from qRT-PCR was expressed as: y = 0.943x + 0.102 (R^2^ = 0.9747). The gene names and the related PCR primers were listed in Tables 1 and 2, The column showed the fold changes (Δ*codY* VS WT) of 6 indicated genes in qPCR. (**c**) The diagram showed the *codY* mutation affected the genes expression related to the metabolic carbohydrates pathway linking to the capsule production and the metabolic amino acid metabolism. ‘↑’ and ‘↓’, the gene expression of Δ*codY* strain was upregulated or downregulated when compared to that of WT strain. (**d**) Electrophoretic mobility shift assay (EMSA) to determine whether purified CodY could bind to promoters of selected virulence regulating genes in *S. suis*. 2. 1,The purified CodY protein at the indicated concentrations was mixed with positive control DNA fragments (PC) and negative control (NC) DNA and subjected to EMSA, respectively. 2,The purified CodY proteins at the indicated concentrations were mixed with indicated promoter DNA fragments (the left upside corner) and subjected to EMSA. The black triangle shows the bands of the protein-DNA complexes. The white triangle shows the free DNA probes. (**e**) The cellular sialic acid contents in *Streptococcus suis* serotype 2 were detected in the wild type (WT) and *codY* mutated (Δ*codY)* strains. The means of two groups were compared using Student’s *t* test (unpaired, 2-tailed). ***P < 0.01.

To understand the mechanism of the *codY* mutation induced decrease in virulence, we isolated the total RNAs of ∆*codY* and WT strain for microarray analysis, followed by comparing the transcript levels of all the genes between the two strains. The bioinformatic analysis afforded by the microarray service company showed that *codY* mutation mainly affected the signals of carbohydrate metabolism, membrane transport, nucleotide metabolism, amino acid metabolism, translation and lipid metabolism (Fig. 6a). Among the 2178 detected genes, the *codY* mutation affected the transcript levels of 404 genes by more than two-fold: 224 were down-regulated and 180 were up-regulated. These regulated genes mainly participate in ABC amino acid transport, physiological metabolism, virulence regulation and capsular synthesis.

To further confirm the validity of the microarray data, quantitative real time PCR (qRT-PCR) analysis using 16S rRNA as an internal standard gene was used to determine the transcript levels for 6 selected genes (Table 1). The data revealed substantial correlations between the results of the microarray analysis and the qRT-PCR (Fig. 6b). According to the microarray assay, the *codY* mutation decreased the expression of virulence related genes, including the capsular polysaccharide assembling related genes including sialic acid synthesis genes, such as sialic acid synthase *neu*A/B/C (Fig. 6c, Table S1) and *cps2J* (SSU05_0573) (Fig. 6b.1,2, Table S1), carbohydrate metabolism related genes including *acc*A/B/C/D (Fig. 6c, Table S1), the muramidase-released protein (MRP) gene, the hemolysin related *sly* (SSU05_1403) gene, adherence related hypothetical protein genes (SSU05_2099, SSU05_2100, SSU05_2101, SSU05_2103) (Table S1). And in the microarray assay *codY* mutation increased the expression of the amino acid metabolism related genes including *ilv*C/D/E/H/I, *leu*A/B/C and *gdh* (Fig. 6c).

Among genes that regulate the virulence of the pathogen, *cps2J*, *codY*, *ilvH* (SSU05_1887), *bap*2 (SSU05_0780), *glo* (SSU05_0725), *glnR* (SSU05_0159), *neuA*, *neuB*, *neuC* (SSU05_0581), *neuD* were selected to investigate whether CodY could bind to their promoters using EMSA. Using a PCR-amplified DNA fragment without a *codY* binding consensus as the negative control and one containing the *codY* binding consensus as the positive control^18^ (Fig. 6d.1), the EMSA assay demonstrated that the purified CodY could bind to the promoters of *bap*2, *cps2J, ilvH, glnR* and *glo* (Fig. 6d.2), which combined with the microarray results, indicated that CodY could directly regulate the expression of these genes. In the EMSA assay, the recombinant CodY did not bind to the promoter of *neuB* (Fig. 6d.2) indicated that CodY could regulate the *neuB* expression not by directly transcriptional pattern.

Meanwhile, the microarray analysis and qRT-PCR demonstrated that the *codY* mutation dramatically affected the expressions of sialic acid synthesis related genes. As one of the main components of the capsule, sialic acid could also affect the adherence of *S. suis* 2. Thus, according to the finding that the *codY* mutation decreased the average capsular thickness of *S. suis* 2 (Fig. 5b), we further detected the sialic acid content in the WT and *∆codY* strains. The result showed that the *∆codY* strain had a significant decrease about sialic acid content (Fig. 6e). This finding could provide a partial explanation of the capsular thickness decrease observed by electron microscopy (Fig. 5b).

## Discussion

In many low G + C Gram-positive bacteria, CodY is global transcriptional regulator that plays a pivotal role in the regulation of microbial metabolism and virulence^33^,^34^,^35^,^36^. Although it has been studied in a number of species, including *Staphylococcus aureus*, *Bacillus subtilis*, *Streptococcus pyogenes* and *Listeria monocytogenes*, the role of CodY in *Streptococcus suis* serotype 2 (*S. suis* 2) has not been investigated in detail.

To better understand the function of CodY in *S. suis* 2, we evaluated the phenotypic change induced by the insertion mutation of *codY*. The *codY* mutation caused a dramatic growth defect in TSB medium, and reduced the cell density of the culture in stationary phase. A previous study showed that mutation of *codY* did not affect the growth rate or growth yield in *Streptococcus pyogenes*^22^. However, the deletion of *codY* may lead to a growth defect when iron becomes a limiting factor for growth^25^. In the present study, the disruption of *codY* inhibited cell growth. Thus, we deduced that the growth regulation by CodY in bacteria might be species or nutritional condition dependent. In Gram-positive bacteria, CodY is considered as a nutrient responsive regulator that binds directly isoleucine^37^. To enrich the understanding of CodY regulating the nutrient response, we evaluate the effect of isoleucine limitation on the growth of WT and *codY* mutant strains. The mutation of *codY* caused a growth flaw in the chemical defined medium with or without isoleucine (Fig. 1g,h). And there was no difference for the growth of WT in presence or absence of isoleucine may because of the compensatory effect when there is isoleucine deficiency. CodY binds directly to isoleucine repressing genes that are required for adaptation to nutrient limitation^37^. Because the *codY* mutation led to the lack of CodY in *S.suis* 2, the binding of CodY to isoleucine could be scarce. This may lead to *codY* mutant strain poorly growing independently on the presence or absence of isoleucine.

Bacterial adhesion is a critical initiation step in bacterial colonization and persistence for pathogens. Bacteria express various adhesive surface structures, such as the capsule, fimbriae or pili, and several surface proteins^38^. Therefore, we assessed the effect of the *codY* mutation on the adhesion and invasion ability of the *S. suis* 2. The *codY* mutation significantly attenuated the adhesion and invasion ability of *Streptococcus suis* serotype 2 (Fig 2a,b), which was consistent with the findings using the macrophage-like RAW264.7 cells (Fig 3a). Although it has been reported that, in the human pathogen *Streptococcus pneumonia*, the *codY* mutation reduced adherence to nasopharyngeal cells^18^, to the best of our knowledge, this is the first study to reveal a function of CodY in regulating the adhesion and invasion ability of *S. suis* 2.

Survival ability in the bloodstream is considered an important factor in the pathogenesis of *S. suis* 2 infection^10^. A previous study revealed that a *codY* mutant of *Staphylococcus aureus* showed reduced survival after phagocytosis^39^. Our bactericidal assays revealed that the *codY* mutant strain showed significantly lower growth in *Cavia porcellus* blood (Fig. 3c), which indicated that CodY is involved in bacterial resistance to phagocytosis. The *in vivo* mouse models revealed that the maximum non-lethal dose (Dn) for wild-type strain was much lower than that of the mutated strain. In addition, the lowest lethal dosage for the wild-type strain was also lower than that of *codY* mutation strain. Further we found that the survival rates were significantly lower in mice infected with the WT strain (2.37 × 10^8^ CFU/mouse) than in that infected with the ∆*codY* strain (3.90 × 10^8^ CFU/mouse) (log-rank (Mantel-Cox) test, P = 0.0247) (Fig. 4a). Combined with the LD_50_ of the wild type strain (2.57 × 10^8^ CFU) and that of mutated strain (1.46 × 10^9^ CFU), we considered that the virulence of ∆*codY* strain was decreased compared with that of the wild-type.

The difference in growth ability in the different tissues or in the blood stream for the wild-type and *codY* mutated strain showed that the growth of *codY* mutated strain was inhibited in different tissues, even being completely eliminated in different tissues at 96 h or 120 h time point in mice (Fig. 4e,f). This result demonstrated that *codY* mutation led to *S. suis* 2 easier being cleared in different tissues even the mechanism is not clear and requires further experimentation.

For *S. suis* 2, the capsule is an important virulence factor^40^; therefore, we examined the morphology of the capsule. We demonstrated that *codY* mutation significantly decreased the capsule thickness in *S. suis* 2 during both exponential phase and stationary phase (Fig. 5b) (Fig. 5a,b). Although CodY has been reported to regulate capsule production^41^,^42^, our results provide direct evidence that CodY regulates capsule synthesis in *S. suis* 2.

The microarray analysis revealed that the *codY* mutation affected important virulence related genes, including capsular polysaccharide synthesis related *cps2J*, muramidase-released protein (MRP) gene, hemolysin related *sly* gene, adherence related hypothetical protein genes and sialic acid synthesis genes such as sialic acid synthase (*neuB*), UDP-N-acetylglucosamine 2-epimerase (*neuC*) and acetyltransferase (*neuD*). The products of *cps2J* and sialic acid synthase regulate capsule synthesis^43^,^44^; therefore, the data suggest a crucial role of CodY in regulating capsule synthesis in *S. suis* 2 (Fig. 5a,b). Combined with the qRT-PCR results, we demonstrated, for the first time, that mutation of *codY* affected the expression of sialic acid synthesis related genes including *csp*2J (Fig. 6b). The sialic acid content test confirmed that the *codY* mutation significantly decreased sialic acid synthesis (Fig. 6e).

Capsular sialic acid is responsible for the binding to virus-infected cell surfaces^45^; Therefore, our finding that the *codY* mutation decreased the sialic acid synthesis in capsule explained both the attenuated adherence and invasion ability of the mutant strain to host cells (Figs 2a,b and 3a). In addition, as a *codY* regulated gene, *neuB* mutation altered the architecture of *S. suis* surface, resulting in attenuated virulence^44^. This, combined with our results, led us to hypothesize that the *codY* mutation in *S. suis* 2 significantly decreased the expression of sialic acid synthesis related genes, resulting in decreased levels of sialic acid in the capsule and reduced capsule thickness, which eventually, at least in part, attenuated the bacterium’s virulence. Though many studies have showed that the decrease of bacterial capsule polysacharides was often accompanied to an increase of adhesion and invasion capacity, Δ*codY* mutation did not increase the adhesion capacity of *S. suis* 2 evern did reduce the capsule thickness (Fig. 5a). The reason might because Δ*codY* mutation affected other factors which induced the attenuation of the adhesion capacity of *S. suis* 2.

In summary, the present study clearly demonstrated that CodY modulates cellular growth and virulence in *S. suis* 2. We also showed that CodY modulates global gene expression, especially genes involved in metabolism and capsule synthesis. Specifically, we provided clear evidence that CodY regulates sialic acid synthesis in the capsule, which is at least partly responsible for the attenuated virulence of *S. suis* 2. In future studies, we will investigate the exact pattern or mechanism of CodY regulating the virulence in *S. suis* 2.

## Methods

### Ethics statement

This study was performed strictly according to the recommendations in the Guide for the Care and Use of Laboratory Animals of Hubei Province, China. The protocol was approved by the Laboratory Animal Monitoring Committee of Huazhong Agricultural University. All efforts were made to minimize suffering.

### Strains, growth conditions and plasmids

The *Streptococcus suis* strain SC19 described in a previous study^2^, was isolated from the brain of dead pigs. The *S. suis* 2 strains were grown in TSB or plated on TSA (Difco Laboratories, Detroit, MI, USA) with 10% (vol/vol) fetal bovine serum at 37 °C. *Escherichia coli* strain DH5*α* was grown in Luria-Bertani broth (LB) liquid medium or on LB agar and used for plasmid construction and propagation. If required, spectinomycin (Sigma, St. Louis, MO, USA) was added to the growth media at 100 μg/mL for *S. suis* 2 and 50 μg/mL for *E. coli*. The cloning vector pMD18-T was from Takara. The expression vector pET-28a was maintained in our laboratory. The temperature-sensitive suicide plasmid pSET4s was kindly provided by Dr Sekizaki (Japan). The shuttle vector pAT18 was from Dr Ulrike M. Samen in Germany.

### Construction of mutant strains

The internal region of *codY*, which does not include the start codon and the helix-turn-helix domain coding sequence, was amplified by PCR using primers *codY’* fw and *codY’* rv (Table 1) and cultured *S. suis* 2 as the template^45^. The PCR product designed as *codY*’ was digested with restriction endonucleases HindIII and EcoRI before being cloned into pSET4s to form the pSET4s–*codY*’ construct. Then the pSET4s–*codY*’ plasmid was transferred to *Streptococcus suis* strain SC19 by electrotransformation, followed by maintaining the transformed cells on TSA plates containing spectinomycin.

To lose the strain containing the plasmids that were not homologous exchanged with the genomic DNA, the obtained clones were grown in TSB culture medium with spectinomycin at 28 °C for 18 hours, followed by re-seeding the cell suspensions at mid-exponential phase at ratio of 1:1000 into the fresh spectinomycin-containing TSB medium for 9–12 hours of culture at 37 °C. The bacterial suspension was then plated TSA plates at 37 °C with spectinomycin and 10% new-born bovine serum and incubated for 12–16 hours. Spectinomycin resistant clones were identified by PCR and the PCR products were sequenced. In the PCR to identify the mutant clones, primers ‘a’ and ‘b’ were designed to overlap the ends of the endogenous *codY* gene, while the primers ‘c’ and ‘d’ were designed against the DNA fragment of the pSET4s–*codY*’ plasmid. The expected PCR product size using primer pair ‘a’/‘b’ was 1334 bp, that using primer pair ‘b’/‘c’ was 1196 bp, that using primer pair ‘a’/‘d’ was 985 bp. In this way, we inserted the entire plasmid into the *codY* gene between the ATG start codon and the region encoding the recognition helix of the conserved helix-turn-helix (HTH) motif responsible for CodY binding to DNA.

### Titration of hemolytic activity

The hemolytic activity was assayed as previously described with modification^46^. Briefly, *S. suis* bacteria were grown in TSB at stationary stage (OD600 0.50–0.75). The supernatant was collected from 1 ml of each culture by centrifugation at 12000 g for 1 min. Serial twofold dilutions (130 μl) of test samples were prepared in polystyrene deep-well titer plates (Corning) with 10 mM PBS (pH 7.4). Subsequently, 130 μl of a 2% washed sheep erythrocyte suspension in 10 mM PBS containing 0.5% BSA was added to each well. Followed the wells being sealed, the plates were incubated on a Coulter mixer for 2 h at 37 °C. Unlysed erythrocytes were sedimented by centrifugation (1500 g for 10 min), 150 μl of the supernatant were transferred to a polystyrene flat-bottom microtiter plate and measured at 540 nm with a microELISA reader. Two different independent assays were carried out in triplicate.

### Analysis of Bacterial Surface Structures

To prepare bacterial surface-associated proteins, bacteria grown in TSB medium were resuspended in 1 ml of 10 mM sodium phosphate (pH 5.5), pelleted by centrifugation at 13,000 × g for 10 min to partially remove proteins not associated with cell surface structures, and then resuspended in 0.2 ml of 10 mM sodium phosphate (pH 5.5). The bacterial suspension was pushed through a 25 G needle four to five times to shear the surface structures and proteins from the bacteria, and then centrifuged at 13,000 × g for 10 min at 4 °C^47^. Twenty microliters of the supernatant was used for SDS-PAGE analysis.

### RNA preparation and Quantitative Real-time PCR (qRT-PCR)

The strains were grown in TSB with 10% (v/v) fetal bovine serum to mid-exponential phase (OD600 ≈ 0.6). Total RNA was isolated using a Promega^TM^ SV total RNA isolation system (Promega), according to the manufacturer’s instructions. RNA concentrations and integrity were determined using an Agilent 2100 Bioanalyzer. The RNA was then used for reverse transcription using PrimeScript^®^ RT master mix (Perfect Realtime) from Takara, also according to the manufacturer’s instructions. The synthesized cDNA was further used for microarray analysis, reverse transcription-PCR (RT-PCR) and quantitative real-time PCR (qRT-PCR).

To confirm the accuracy of the microarray data by qRT-PCR using SYBR Green detection, 16S rRNA was used as the reference gene. The qRT-PCR was conducted using the THUNDERBIRD SYBR qPCR Mix (Toyobo, Japan) according to the manufacturer’s instructions. Quantitative analysis was performed in triplicate with an ABI 7500 Fast Real-Time PCR system. The relative expression level was calculated using the comparative cycle threshold (2^−ΔΔCt^) formula normalized to the 16S rRNA level. Student’s *t* test was performed to verify the significance of the qRT-PCR. The sequences of primers are shown in Table 1.

### Western blot analysis

Samples of supernatant fluid were loaded on a 12% SDS-PAGE after boiling in sample buffer containing 20 mM dithiothreitol (DTT) for 10 min at 95 °C. The separated proteins were then electroblotted onto a PVDF membrane. The primary antibody was immunoreactive rabbit serum raised against purified His-tagged CodY. The secondary antibody was goat anti-rabbit antibody. Diaminobenzidine dissolved in 50 mM Tris (pH 7.2) at 1 mg/mL (immediately added with an equal volume of 0.02% hydrogen peroxide) was used to visualize the immunoreactive protein bands.

### Purification of His-tagged CodY protein and Electrophoretic Mobility Shift Assays (EMSA)

The *codY* gene was amplified from the *S. suis* 2 genomic DNA by PCR using primers his-*codY*1 fw and his-*codY*1 rv (Table 1). The PCR product was cloned into pET28a to produce pET28a-*codY*-His, which was transformed into the *E. coli* strain BL21(DE3). The transformed BL21 strain was grown to mid-exponential phase (OD600 ≈ 0.6) in LB containing spectinomycin and then induced with 1 mM/mL isopropyl β-D-1-ihiogalactopyranoside (IPTG) for 3 h. The strains were collected by centrifugation, and the 6-His-tagged CodY protein was purified using nickel ion affinity chromatography (Novagen). The concentration of CodY was determined in comparison with bovine serum albumin as the standard.

The native purified CodY protein (0 ~ 2200 ng) was used for EMSA. The probes for EMSA were obtained by PCR amplification using the primers shown in Table 2. The DNA probes for positive control and negative control were obtained by PCR using genome of *Streptococcus pneumoniae* as the template^18^. The PCR products were verified by sequencing and 1 μg was used to form a DNA-protein complex in binding buffer (Beyotime Company, China) for 30 min at 37 °C. The DNA-protein complex was loaded onto a native PAGE gel without SDS. After electrophoresis, the gel was stained with ethidium bromide followed by imaging.

### Phagocytosis of *S. suis* 2 mediated by RAW 264.7 macrophages and bactericidal assays using fresh Cavia porcellus blood cells

Murine macrophage-like RAW264.7 cells were cultured in DMEM supplemented with 10% FBS. The wild-type and *codY* mutant strain were incubated with RAW264.7 macrophages at a bacteria-to-cell ratio of 100:1, separately. After co-cultured with the *S. suis* 2 for 1 h, the macrophages were washed three times with PBS and then incubated in DMEM media containing ampicillin (100 μg/ml). After 1 h and 2 h of incubation, the macrophages were sampled, washed three times with PBS and lysed with water. Serial dilutions of the lysates at each time point were plated on TSB agar. The number of colonies formed in each group at 1 h and 2 h time points was determined. The phagocytosis of *S. suis* 2 by RAW264.7 cells was obtained at time point of 1 h and 2 h. Each assay was performed in triplicate.

In the bactericidal assays conducted as previous^2^, 100 μl of the indicated strains at logarithmic phase were diluted 10-fold in PBS without incubation (termed as the 0 h time point) or by fresh whole *Cavia porcellus* blood and incubated for 2 hours (termed as the 2 h time point). The *S. suis* 2 contained suspensions from each treatment for the wild-type and *codY* mutant strain were plated at 10^−5^ and 10^−6^ dilutions on TSA plates, respectively. The obtained CFUs were counted for wild-type and *codY* mutant group at 0 h and 2 h time points. Because there was net growth for both wild-type and *codY* mutant strains in blood, we designed growth factors as the ratio of CFU in each sample after 2 h incubation over the CFU in the corresponding inoculum^2^. Each assay was performed in triplicate.

### Adherence and invasion assay using HEp-2 cells

Adherence of *S. suis* 2 to the HEp-2 cell model has been described previously^30^. In brief, HEp-2 cells were seeded into 24-well tissue culture plates at 10^5^ cells per well and cultivated overnight in DMEM culture medium with 10% fetal calf serum (Gibco). The cells were infected with *S. suis* 2 (10^6^ CFU) at a multiplicity of infection (MOI) of 10:1 and incubated at 37 °C for 2 h. The infected cells were then washed three times with PBS. The number of cell-adherent bacteria was determined three times in each group by lysing the cells using sterile distilled water, plating appropriate dilutions of the lysates on TSA and calculating the CFU. The TSA agar with *S. suis* 2 was cultivated at 37 °C for 24 h, to calculate the number of bacteria that had adhered to cells. The adherence experiments were repeated at least three times.

To assay *S. suis* 2 invasion into HEp-2 cells, *S. suis* 2 (10^6^ CFU) at an MOI of 10:1 was incubated with HEp-2 cells at 37 °C for 2 h. The infected cells were washed three times with PBS before being kept in DMEM culture medium containing gentamicin (100 μg/ml) and penicillin (5 μg/ml) for 2 h to kill the bacteria remaining outside the HEp-2 cells. The HEp-2 cells were then washed three times by PBS, followed by lysing the cells using sterile distilled water, plating appropriate dilutions of the lysates on TSA and calculating the CFU. Each assay was performed in triplicate.

### Mouse infections

For survival curves of mice infected with *S. suis* 2 strains, 10 female BALB/c mice (6-weeks-old) in each group (group I, II, III, IV) were inoculated intraperitoneally with the indicated CFU in 200 μL PBS for the wild-type (WT) or mutated *codY* (Δ*codY*) strain. Groups I and II were the lower dose groups, which were inoculated with 1.18 × 10^8^ CFU of the WT and 3.90 × 10^8^ CFU of the Δ*codY* strain for each mouse, respectively. Groups III and IV were the higher dose groups, which were inoculated with 2.37 × 10^8^ CFU of the WT and 9.75 × 10^8^ CFU of the Δ*codY* strain for each mouse, respectively. Ten female mice in group V were injected with 200 μL PBS for each mouse as the control group. Mice were monitored daily for 11 days to determine survival rates.

To further investigate the elimination of *S. suis* 2 in mice tissues, we infected 30 mice using the WT and Δ*codY* strain at dose of 5 × 10^7^ CFU/mouse respectively. On time point of 18, 24, 48, 72, 96, 120 and 240 h post-infection, three mice in each strain were sacrificed followed by collecting the blood, heart, brain and lung samples. Then the heart, lung and brain were weighed and homogenized in 1 mL PBS. Homogenates were serially diluted and plated on TSA with (for WT) or without spectinomycin (for the mutant strain) to determine the number of viable bacteria. Blood samples were directly diluted for plating.

### Colony morphology analyzed by transmission electron microscopy

To evaluate the morphological changes in the Δ*codY* strain, transmission electron microscopy (TEM) was conducted according to previously reported methods^48^. Briefly, the bacteria from mid-exponential phase were fixed in 5% glutaraldehyde for more than 2 hours. The samples were dehydrated and embedded in epoxy resin before being sectioned. The sections were examined under TEM (Tecnai) at an accelerating voltage of 200 kV. The Image J software was used to determine capsule thickness by measuring the cross-sectional areas of randomly chosen bacteria, including and excluding the capsule. Assuming circularity, the areas were then used to calculate the capsule layer width. The experiments were performed 3 times independently. In each time, the average capsule thickness was obtained for 35 randomly chosen bacteria of each strain investigated. And the representative images of each strain from one experiment was showed. Then for each strain, the statistical capsule thickness was showed using the average of 3 average values from 3 independent experiments with 35 determinations.

### DNA microarray-based analysis

DNA microarray analysis was performed using an Agilent custom-designed oligonucleotide microarray. Based upon the whole genome sequence of 05ZYH33^49^, specific 60-mer oligonucleotide probes were designed using eArray (https://earray.chem.agilent.com/earray/) to cover nearly all annotated genes. Probes were printed seven times on the microarray slides. Two biological replicates of total RNA from the WT strain and *codY* mutant strain were amplified and labeled with Cy3-CTP using a Low Input Quick Amp Labeling Kit, one-color (Agilent technologies, US), following the manufacturer’s instructions. Labeled cRNA was purified using an RNeasy mini kit (Qiagen). After fragmentation, the microarray slides were hybridized with 600 ng of Cy3-labeled cRNA. Hybridization was performed at 65 °C for 17 h with rotation at 10 rpm. The microarray slides were washed and scanned using an Agilent Microarray Scanner (G2565BA). Those genes with greater than two-fold change ratios were regarded as differentially expressed genes. Microarray data has been deposited into the NCBI Gene Expression Omnibus (GEO) with accession number GSE68068 (http://www.ncbi.nlm.nih.gov/geo/query/acc.cgi?acc=GSE68068).

### Data analysis

Survival data were analyzed with the log-rank (Mantel-Cox) test. Other data are expressed as means ± *S.D*. The means of two groups were compared using Student’s *t* test (unpaired, 2-tailed), with *P* < 0.05 considered to be statistically significant. Unless indicated in the figure legends, all the experiments were performed at least three times with similar results. Statistical analysis was performed using GraphPad Prism 5 (San Diego, USA).

## Additional Information

**How to cite this article**: Feng, L. *et al*. The CodY regulator is essential for virulence in Streptococcus suis serotype 2. *Sci. Rep*. **6**, 21241; doi: 10.1038/srep21241 (2016).

## Supplementary Material

Supplementary Information

## Figures and Tables

**Table 1:** List of primers used in this study.

Primer	Sequence	Remark
**Cloning**		
* codY’* fw	TTTTAAGCTTGAAGAGCAATTGGCAGAAGAA	*Hin*d III
* codY’* rv	TTTTGAATTCGCAATCACAGAAGCAGTCAAT	*Eco*R I
* *‘a’: *codY*test1	CATCAGTCGTATAGGTCAGAT	/
* *‘b’: *codY*test2	AATGACGTTTATCCATCCAAC	/
* *‘c’: pSET4Stest2	AATGACGTTTATCCATCCAAC	/
* *‘d’: pSET4Stest1	TCGCTATTACGCCAGCTG	/
* *X-f:	TCTCTACACAAAGTGGGCTACC	
* *X-r:	GACTGGCTCGCAACCTTCTT	
* *Y-f:	CCGATAGAAGAAGCAGCCTCT	
* *Y-r:	ACGGCACACTTAAATCCTTTGA	
**CodY Overexpression**		
* *his-*codY*1 fw	TTTTCCATGGGCCATCATCATCATCATCACAGCATGACAACATTATTAGAGAAGACAC	*Nco* I
* *his-*codY*1 rv	TCTTGGATCCTTAGTAGTCACGTTTCTTAATTTCAT	*Bam*H I
* ***qRT- PCR**		
* ilv*H-F	GAGTGAGGTGCTACATCGACAACAT	SSU05_1887
* ilv*H-R	AACAAATCATCAAACAGCTAAACCG	
* bap*2-F	CTTTACGACGACGGCTGGCTTGATT	SSU05_0780
* bap*2-R	GTGATGATATTGTTCAAACCGAGGT	
* glo*-F	CCAATCATCGAAACGGAACCAATC	SSU05_0725
* glo*-R	TATGATAGGTCAAGCCAAGCAACTG	
* gua*A-F	CCTTTTTCGGCGGTGGACAGGACTA	SSU05_1472
* gua*A-R	TAATATTGACCGAGCAACTGGAAGC	
* acc*-F	TGGCTTCAATGGGCACAGTGGT	SSU05_1797
* acc*-R	GTAAAGAGGACGACTGGCAAAC	
* cps*2J-F	ACGCAGAGCAAGATGGTAGAATAAAA	SSU05_0573
* cps2*J-R	CAAGTAACCCTCCCGACAAATCACTAT	SSU05_0573
* *16S RNA-F	ATGGACCTGCGTTGTATTAGC	
* *16S RNA-R	CATTGCCGAAGATTCCCTAC	

*Underlined regions in the primers represent the cleavage sites for the restriction enzymes mentioned in the remarks column; qRT- PCR, Quantitative Real Time PCR.

**Table 2:** PCR Primers used to generate EMSA probes.

Primer	Sequence	Remark
Positive Control EMSA F	CCAAAAACTGAATTGAAAGAATTT	gdhA promoter, Ref. 18
Positive Control EMSA R	CTTTAGCAGATGTCATATCGTTCTCC	
Negative Control EMSA F	TGAAAGAAGAGCTATTTTCGTCAT	psaR promoter, Ref. 18
Negative Control EMSA R	CTTTGTTTGGGGTCATTCGT	
*bap*2 EMSA F	ATCTCGGTCTTTGGTTGTTT	SSU05_0780
*bap*2 EMSA R	AGGTATTTACTGCTACGACACTAA	
*cps*2J EMSA F	TCTGTATTTATCATTGTAGTCCTAT	SSU05_0573
*cps*2J EMSA R	TGCTATCATAAAAATCATCA	
*ilv*H EMSA F	TGAACTTCACCCCCAACATT	SSU05_1887
*ilv*H EMSA R	CCGATTAAGGTTGTGTTGAT	
*gln*R EMSA F	GGAGTTTTCATTGCGATTCT	SSU05_0159
*gln*R EMSA R	AATACAAGCGACGATTCCCT	
*glo* F	TTTTGGTCTTGGGATTACTG	SSU05_0725
*glo* R	AAAAACTTCCCTAACCATTG	
*neu* EMSA F	GGACGTATGCGCCTCTGCTT	SSU05_0578
*neu* EMSA R	AAATGACTGTAAATCGCTCCC	

*Note: *neu* EMSA primers were overlapped the promoter of *neuA*, *neuB*, *neuC* and *neuD*.
